# The value of heparin-binding protein in bronchoalveolar lavage fluid in acute respiratory distress syndrome

**DOI:** 10.3389/fmed.2025.1537680

**Published:** 2025-02-17

**Authors:** Yang Liu, Wei Zhou, Wei Xu, Xing-ping Lv, Fei-fei Wang, Xue-bin Wang, Shao-lin Ma

**Affiliations:** Department of Critical Care Medicine, Shanghai East Hospital, Tongji University School of Medicine, Shanghai, China

**Keywords:** heparin-binding protein, bronchoalveolar lavage fluid, ARDS, prognosis, ALI/ARDS

## Abstract

**Background:**

Heparin-binding protein (HBP) is recognized as a significant factor in the development of Acute Respiratory Distress Syndrome (ARDS). Although plasma levels of HBP have been identified as a predictive biomarker for ARDS, the role and value of HBP in bronchoalveolar lavage fluid (BALF) remain unexplored.

**Methods:**

Our study utilized a cecum ligation and puncture (CLP) method to induce an ARDS model in mice, examining the correlations between plasma and BALF HBP levels, lung injury severity, lung wet-to-dry (WD) ratio, and BALF total protein levels. Additionally, we conducted a comparative analysis of BALF and plasma HBP levels in 44 ARDS patients and 38 patients with cardiogenic pulmonary edema (CPE), investigating their correlations.

**Results:**

In the animal study, CLP-induced mice demonstrated significantly higher lung WD ratios, BALF protein, BALF HBP, and plasma HBP levels compared to the control group. Notably, both BALF and plasma HBP levels were significantly correlated with lung injury severity. In human subjects, significant differences in BALF HBP, BALF protein, and plasma HBP levels were observed between ARDS and CPE patients, along with notable correlations between these markers and the severity of lung injury. Particularly, BALF HBP levels exhibited a stronger correlation with lung injury compared to plasma HBP levels.

**Conclusion:**

The study indicates that both BALF and plasma HBP levels are significantly elevated in the context of lung injury in both animal models and human ARDS patients. More importantly, BALF HBP levels show a stronger correlation with the severity of lung injury, suggesting that BALF HBP could serve as a valuable biomarker for diagnosing and guiding the treatment of ARDS.

## 1 Introduction

Acute respiratory distress syndrome (ARDS) presents a critical challenge in intensive care units globally, characterized by sudden respiratory failure. Despite extensive research, the precise mechanisms underlying ARDS remain partially understood. It is commonly accepted that ARDS represents a severe form of the systemic inflammatory response syndrome (SIRS) manifesting within the lungs. The condition is marked by the damage to pulmonary vascular endothelial cells and alveolar epithelia, which triggers an overwhelming inflammatory response. This results in increased capillary permeability, allowing fluid and proteins to leak into the alveolar and interstitial spaces, thereby compromising lung function. Understanding the dynamics of pulmonary vascular permeability is, therefore, pivotal in ARDS research.

Neutrophils are key players in modulating capillary permeability, with their accumulation and subsequent cytokine release being critical in the pathogenesis of ARDS, contributing to alveolar capillary damage and enhanced permeability (1). Among the potent inflammatory mediators released by activated neutrophils is the heparin-binding protein (HBP), which is a versatile modulator of inflammation. It promotes monocyte recruitment, adhesion, and transmigration (2), serving as a vital signal to vascular endothelial cells and playing a significant role in the vascular leakage and pulmonary edema characteristic of ARDS (3). In sepsis cases, plasma HBP levels are significantly elevated and associated with the development of hypotension and organ dysfunction. Rapid assessment of HBP concentration is valuable for early diagnosis and prognostic evaluation of severe sepsis (4).

Clinical investigations have demonstrated a significant elevation in plasma HBP levels in ARDS patients, correlating with the syndrome’s onset (5). Plasma HBP levels of ALI/ARDS patients were significantly higher than that of CPE patients. HBP was a strong prognostic marker for short-term mortality in ALI/ARDS (6). In animal studies of ventilator-associated lung injury, although the increase in HBP in plasma was not significant compared to the control group, there was a significant increase in HBP in BALF, which gradually increased over time (7). HBP is implicated as a critical effector in the etiology of transfusion-related acute lung injury and sepsis-induced ARDS (8). Our previous research has established a concurrent rise in neutrophil counts and HBP levels in ARDS cases, highlighting HBP’s predictive value for the syndrome’s development. Furthermore, we have identified the β2 integrin-PI3K signaling pathway as a key mechanism in HBP release from PMNs, offering new avenues for therapeutic intervention (9).

Despite the recognized significance of plasma HBP levels, the investigation into bronchoalveolar lavage fluid (BALF) HBP and its connection to ARDS is lacking. Given that BALF directly samples the alveolar surface, it may contain higher concentrations of cytokines, including HBP. HBP levels in the BALF of lung injury patients have not previously been studied, and whether HBP plays a role in VILI has, to our knowledge, not been studied previously. We hypothesize that BALF HBP levels are significantly elevated in ARDS patients, aiming to explore its levels and implications in both animal models of acute lung injury (ALI) and human ARDS cases. By examining HBP levels in BALF and blood in a CLP-induced rat model of ALI and comparing these markers in ARDS patients and individuals with cardiogenic pulmonary edema, we seek to elucidate BALF HBP’s role in ARDS. The assessment of lung injury severity through the wet/dry lung weight ratio and BALF total protein levels further complements our investigation.

## 2 Methods

### 2.1 Animal studies

#### 2.1.1 Animals

Male C57BL/6 mice aged 6–8 weeks and weighing 22–25 g were sourced from the Experimental Animal Centre of Tongji University Medical College, Shanghai, China. These mice were accommodated in a pathogen-free environment with wood shavings, under a controlled 12-h light cycle, and had unrestricted access to food and water. All procedures adhered to international, national, and institutional guidelines for animal care and use.

#### 2.1.2 Grouping and treatment

We divided 24 mice randomly into two groups: sham and cecum ligation and puncture (CLP). Mice in the CLP group underwent the CLP procedure, whereas Sham group mice underwent a similar procedure without cecal ligation or puncture.

#### 2.1.3 Cecal ligation and puncture procedure

Under anesthesia with 1.5% pentobarbital sodium, a 1 cm abdominal incision was made to expose the cecum, which was then ligated below the ileocecal junction with 4-0 silk and punctured using an 18-gauge needle. A small amount of fecal material was expelled to verify puncture patency before the cecum was repositioned and the incision sutured. Within approximately 6–12 h after the CLP procedure in mice, the lungs begin to show signs of acute injury, including shortness of breath, cyanosis around the mouth, reduced activity, and refusal to eat. The above symptoms worsen further within about 24 h, and there is a peak period of death within 24–48 h. No mechanical ventilation was given to the mice during the operation.

Twenty-four hours after CLP or sham operation, mice were anesthetized for plasma HBP extraction and bronchoalveolar lavage. The left lungs were reserved for wet/dry weight ratio analysis.

#### 2.1.4 Bronchoalveolar lavage

Anesthetized mice had an 18-gauge catheter inserted into the right bronchus for PBS instillation (0.5 mL). This process was repeated twice, pooling effluents for protein quantification. Half of the bronchoalveolar lavage fluid (BALF) was allocated for protein measurement via BCA Protein Assay, while the remainder was frozen at −80°C for HBP analysis.

#### 2.1.5 Blood and BALF HBP measurement

Blood was collected from an arterial line into EDTA vacutainer tubes and immediately chilled and centrifuged at 2,000 RCF for 10 min. The plasma was then stored at −80°C until HBP measurement.

To address changes in HBP and protein concentration levels caused by variability in BALF sampling, we used urea dilution method to correct HBP and protein concentration. The urea dilution method utilizes the principle that urea in plasma can freely diffuse into the alveoli to measure the concentration of urea in plasma and BALF, calculate how many times the lavage fluid has been diluted, and use this multiple to correct the protein concentration in the lavage fluid. All BALF and plasma were simultaneously measured for urea levels.

The calculation formula is: corrected concentration = uncorrected concentration * plasma urea concentration/BALF urea concentration. All statistical analyses were performed using the corrected protein concentrations.

#### 2.1.6 Wet/dry lung weight ratio

To assess pulmonary edema, the left lung was weighed for its wet weight, dried at 55°C for 48 h for dry weight, and the wet/dry ratio calculated.

#### 2.1.7 Enzyme-linked immunosorbent assay

HBP levels in plasma and BALF were quantified using an Azurocidin (HBP) Enzyme-linked immunosorbent assay (ELISA) kit (Boster, Wuhan, China) as per the manufacturer’s instructions. Briefly, 100 μL of each sample was added to anti-human HBP antibody-coated wells, incubated, followed by the addition of a biotin-conjugated secondary antibody and subsequent incubation steps. After adding the avidin-biotin complex and tetramethylbenzidine (TMB), the reaction was stopped and read at 450 nm on a microtiter plate reader to determine HBP concentrations.

### 2.2 Human studies

We embarked on a prospective observational study to examine the significance of bronchoalveolar lavage fluid (BALF) HBP in patients with acute respiratory distress syndrome (ARDS), using individuals with cardiogenic pulmonary edema (CPE) as a comparator. The main endpoint of the study is to determine whether there is a significant increase in HBP in BALF of ARDS patients. The secondary endpoint is to determine whether the elevated level of HBP in BALF is consistent with the severity of lung injury in patients, and whether it is consistent with the elevated protein in bronchoalveolar lavage fluid.

### 2.3 Study population

The study included patients admitted to the intensive care units (ICUs) of Tongji University Shanghai East Hospital from May 2018 to October 2020, who were screened for ARDS onset. Eligible participants were those aged 18 to 89 years, diagnosed with ARDS, underwent fiberoptic bronchoscopy in the ICU, and provided informed consent. Exclusion criteria encompassed pregnancy, history of malignant tumors, post-cardiopulmonary resuscitation status, supporting with mechanical circular support, chronic interstitial lung disease or COPD, pulmonary embolism, immunosuppression due to medication or disease, severe renal insufficiency and ongoing hemodialysis. Prior to the study, sample size estimation was conducted based on the preliminary experimental results. At least 35 patients were included in each group, and the recruitment target was ultimately targeted at 35–45 patients. All patients in the CPE group were those who received endotracheal intubation and mechanical ventilation due to cardiogenic pulmonary edema which is not induce by infection. All patients with ARDS were treated with low tidal volume ventilation, low plateau pressure, and appropriate PEEP (set using the optimal compliance method). After intubation, appropriate sedation was administered. If there was obvious respiratory distress (such as respiratory rate >30 times/min), muscle relaxants were used for treatment. Patients with CPE were supported with PEEP levels of 5–10 cmH₂O.

ARDS patients met the criteria of the ARDS Berlin definition, which include: (1) onset within 1 week of a known clinical insult or new/worsening respiratory symptoms; (2) bilateral opacities on chest radiograph or CT scan not fully accounted for by effusions, lobar/lung collapse, or nodules; (3) categorized as mild (PaO_2_/FiO_2_ >200 mmHg and ≤300 mmHg with PEEP or CPAP ≥5 cm H_2_O), Moderate (PaO_2_/FiO_2_ >100 mmHg and ≤200 mmHg with PEEP or CPAP ≥5 cm H_2_O), or Severe (PaO_2_/FiO_2_ ≤ 100 mmHg); and (4) respiratory failure not solely attributable to cardiac failure or fluid overload, or objective assessment to rule out hydrostatic edema if no risk factor is present.

### 2.4 BALF and blood HBP measurement

Fiberoptic bronchoscopy was performed on all patients within 24 h of enrollment. During the procedure, patients were intubated and ventilated. A 15-min preoxygenation was carried out before the bronchoscope was inserted through the tracheal tube into a bronchus of the middle or lingual lobe. Three aliquots of 20 mL of sterile 0.9% NaCl at room temperature were instilled (totaling 60 mL) and subsequently recovered via gentle suction. The lavage fluid was processed and half of the supernatant was used for BALF protein analysis using the BCA Protein Assay kit protocol, while the remainder was preserved at −80°C for HBP level assessment.

Blood samples were drawn from arterial lines into EDTA vacutainer tubes, immediately chilled, and centrifuged at 3,500 rpm for 10 min. The plasma obtained was then stored at −80°C until further analysis.

HBP concentrations in both BALF and plasma were quantified using an enzyme-linked immunosorbent assay (ELISA).

### 2.5 P/F ratio calculation

Arterial blood gas analysis was conducted on all patients before the bronchoscopy to calculate the P/F ratio, defined as the ratio of arterial oxygen partial pressure (PaO_2_ in mmHg) to the fraction of inspired oxygen (FiO_2_, expressed as a percentage).

### 2.6 Statistical methods

Statistical analyses were performed using SPSS version 26.0 and GraphPad Prism 9 software. For categorical variables, differences were assessed using Fisher’s exact test. Quantitative data are presented as mean ± standard deviation (SD), and comparisons between groups were conducted using the independent samples *t*-test. The S–W test is used to verify whether the data is normally distributed, and logarithmic comparison is performed on non-normally distributed data. Correlation analyses were carried out employing the Pearson method. The association between plasma and BALF HBP levels was examined through logistic regression analysis. A *p*-value of less than 0.05 was considered statistically significant.

## 3 Results

### 3.1 Animal studies

**Figure 1:**
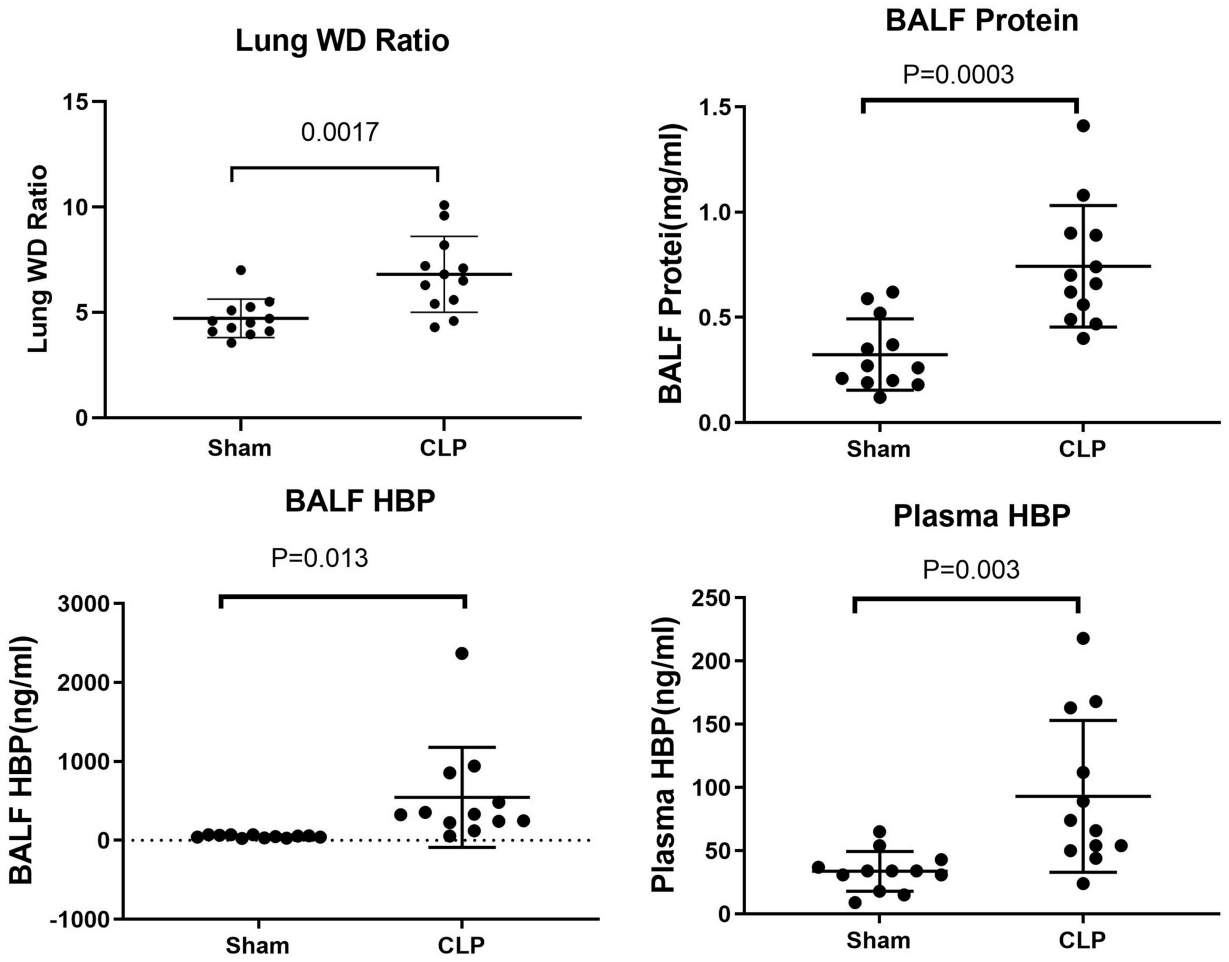
Comparison of lung WD ratio, BALF protein, BALF HBP, and plasma HBP between sham and CLP group mice.

In comparison to the sham group, mice in the CLP group exhibited significantly higher lung wet/dry (WD) ratios (4.72 ± 0.26 vs. 6.81 ± 0.52, *p* = 0.0017) and bronchoalveolar lavage fluid (BALF) protein levels (0.32 ± 0.05 vs. 0.74 ± 0.08, *p* = 0.0003). Similarly, levels of BALF HBP (50.75 ± 5.17 vs. 545.81 ± 182.92, *p* = 0.013) and plasma HBP (33.75 ± 4.53 vs. 93.00 ± 17.34, *p* = 0.003) were also significantly elevated in the CLP group compared to the sham group (see Figure 1).

**Figure 2:**
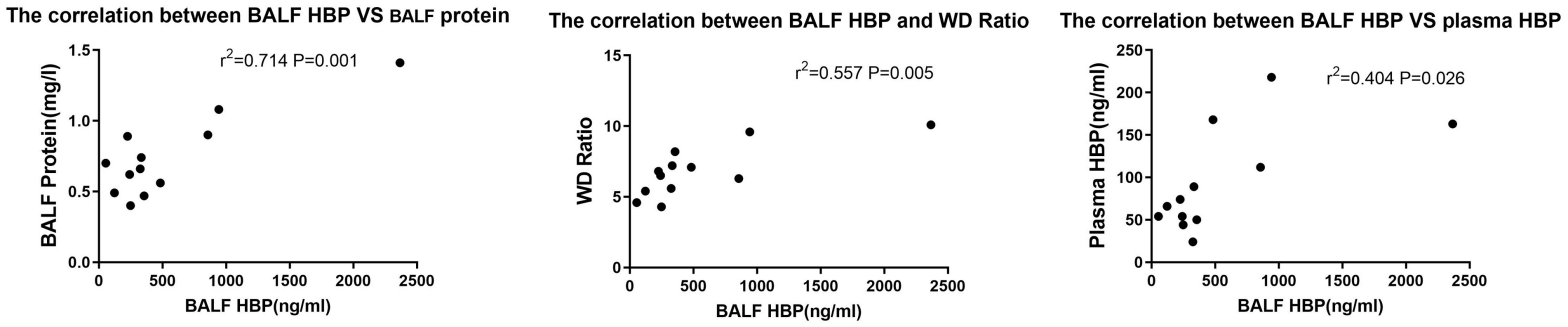
Correlation between BALF HBP, plasma HBP, and P/F ratio in CLP group mice.

The BALF protein and lung WD ratio is considered an important indicator of the severity of lung injury. We conducted a correlation analysis between animal BALF HBP with BALF protein and WD ratio to clarify the relationship between HBP and lung injury. Previous studies have suggested that plasma HBP is elevated during ARDS, so the correlation of BALF HBP and plasma HBP were also analyzed. Analysis revealed a significant correlation between BALF HBP with BALF protein (*r*^2^ = 0.714, *p* = 0.001) and WD ratio (*r*^2^ = 0.557, *p* = 0.005) in CLP group mice. Additionally, a significant correlation was observed between plasma and BALF HBP (*r*^2^ = 0.404, *p* = 0.026) (see Figure 2).

#### 3.1.1 Characteristics of the study population

**Figure 3:**
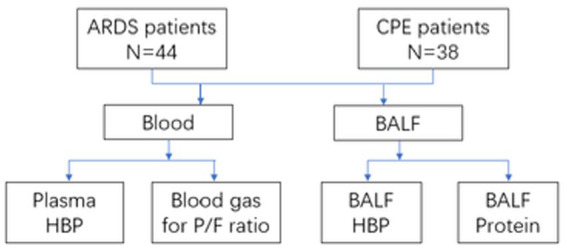
The process of obtaining patient research data.

Ultimately, 44 ARDS patients and 38 cardiogenic pulmonary edema (CPE) patients who were intubated and mechanical ventilation in ICU were included in the study (see Figure 3). Necessary vasoactive drugs were used to maintain patients’ mean arterial pressure above 65 mmHg. The patients had oxygen saturation above 90% 25 with the support of a ventilator, and PEEP was used appropriately according to their condition. The patients were given appropriate sedation. After completing the signing of the informed consent form, patients drew blood samples within 24 h after a clear diagnosis was made, and underwent fiberoptic bronchoscopy examination.

Both groups had comparable age (mean age 60.5 vs. 55.6 years) and gender distribution (male 54.5% vs. 52.4%). In the ARDS group, there were 10 cases (22.7%) of abdominal infection, 6 cases (13.6%) of urinary infection, 24 cases (54.5%) of pulmonary infection, 4 cases (9.1%) of soft tissue infection, and 2 cases of blood transfusion. The CPE group consisted of patients with cardiogenic pulmonary edema and hypoxic endotracheal intubation. There was no significant difference in age, gender and ventilator drive pressure between the two groups of patients. Except for CPE patients who have a higher history of coronary heart disease, there is no significant difference in other medical histories (see Table 1).

**Table 1:** Patient characteristics.

	ARDS group	CPE group	*F*/*t*	*p*
Patients, *n*	44	38		
Gender, % male	24/20	20/18	0.020	0.89
Age (mean ± SD)	60.5 ± 13.3	55.6 ± 12.4	7.86	0.76
Medical history				
Hypertension	20	24	2.570	0.109
Diabetes mellitus	12	16	1.995	0.158
Coronary heart disease	29	34	6.360	0.012
COPD	1	1	0.011	0.916
Chronic renal insufficiency	2	4	1.076	0.300
P/F ratio				
200–300 mmHg	21	/		
100–200 mmHg	12	/		
<100 mmHg	11	/		
Parameters of ventilator				
Tidal volume (mL)	393.9 ± 36.5	411.3 ± 50.6	1.806	0.075
Respiratory rate (breaths/min)	30.1 ± 4.9	31.6 ± 5.3	1.313	0.193
Driving pressure (cmH_2_O)	12.8 ± 1.3	12.6 ± 1.7	0.582	0.562
Positive end expiratory pressure (cmH_2_O)	9.2 ± 2.7	8.2 ± 1.4	1.947	0.055
Peak pressure	22.3 ± 2.5	20.9 ± 2.1	0.523	0.643
Etiology of ARDS, *n*				
Intra-abdominal infection	10			
Urinary system infection	6			
Lung infections	24			
Soft tissue infection	4			
Blood transfusion	2			
Severity of ARDS, *n*				
PaO_2_/FiO_2_ 200–300 mmHg	21			
PaO_2_/FiO_2_ 100–200 mmHg	12			
PaO_2_/FiO_2_ <100 mmHg	11			

Data are presented as the median ± standard deviation.

#### 3.1.2 Comparison between two groups

**Figure 4:**
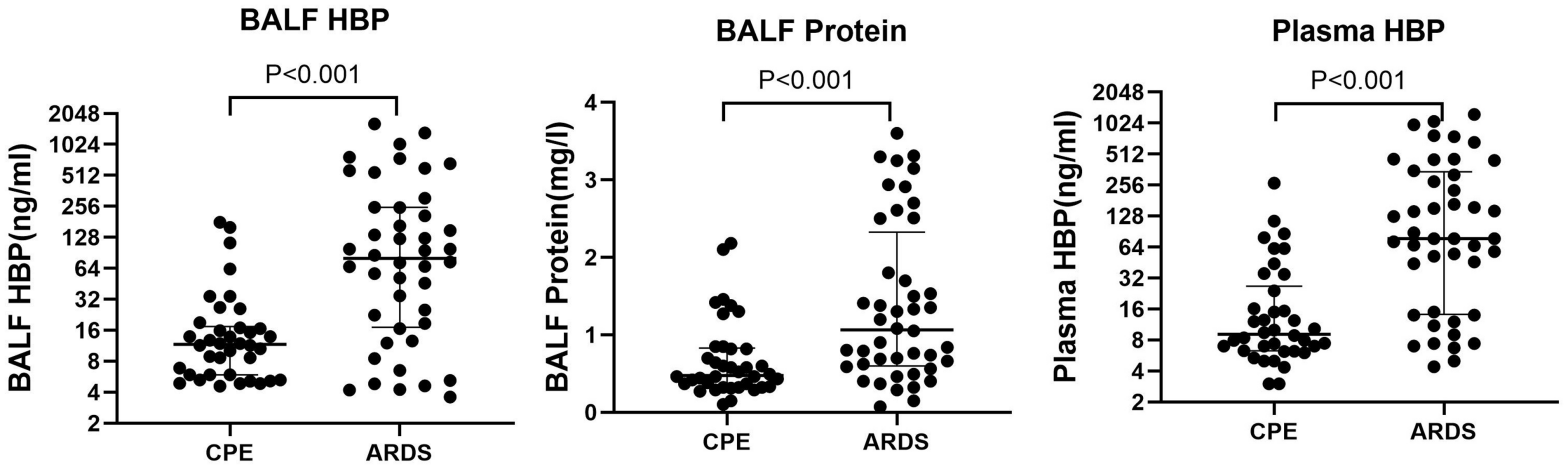
Comparison of BALF HBP, BALF protein, and plasma HBP between ARDS and CPE groups.

The levels of BALF HBP and plasma HBP between the ARDS and CPE groups were compared. There were significant differences in BALF HBP (24.532 ± 39.349 vs. 240.583 ± 366.403, *t* = 6.298, *p* < 0.001), BALF protein (0.675 ± 0.492 vs. 1.387 ± 1.017, *t* = 5.846, *p* < 0.001), and plasma HBP (27.403 ± 47.175 vs. 233.180 ± 310.502, *t* = 7.034, *p* < 0.001) between the two groups (see Figure 4).

#### 3.1.3 BALF HBP and lung injury severity in patients

**Figure 5:**
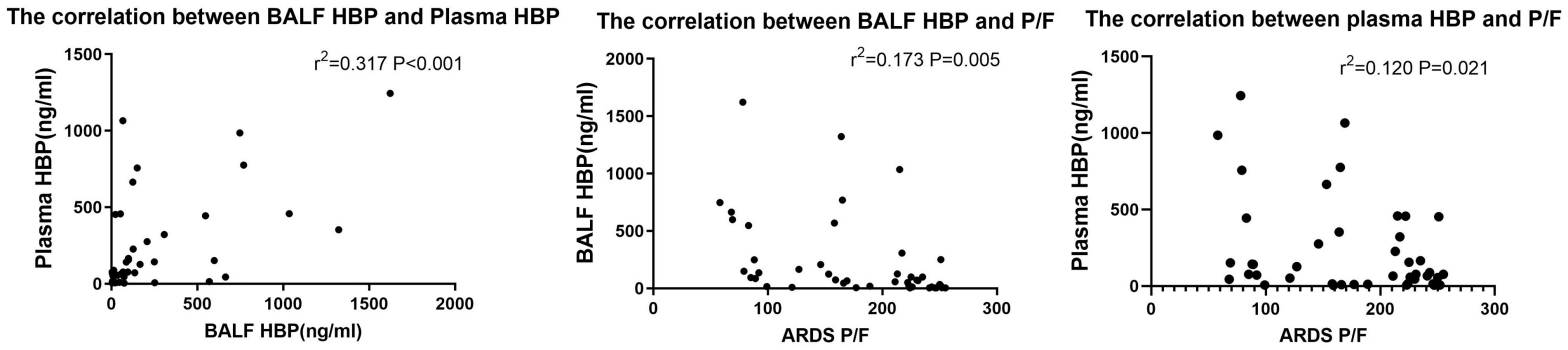
Correlation between BALF HBP, plasma HBP, and P/F ratio in ARDS patients.

The P/F ratio is considered a severity index of lung injury. To clarify the relationship between HBP in BALF and plasma HBP with the degree of lung injury, we analyzed their correlation with P/F index. Results showed there were P/F ratio have significant correlations with BALF HBP (*r*^2^ = 0.173, *p* = 0.005) and plasma HBP (*r*^2^ = 0.120, *p* = 0.021). Additionally, a significant correlation was found between BALF HBP and plasma HBP (*r*^2^ = 0.317, *p* < 0.001) (see Figure 5).

## 4 Discussion

While the precise mechanisms underlying acute lung injury (ALI) and acute respiratory distress syndrome (ARDS) remain to be fully elucidated, the prevailing view among scholars is that these conditions are predominantly driven by a systemic inflammatory response syndrome (SIRS) triggered by infection. In response to infectious stimuli, the immune system releases a vast array of inflammatory mediators, leading to neutrophil degranulation, the release of proteolytic enzymes, and increased capillary endothelial cell permeability. This sequence of events facilitates the leakage of fluid and protein into the interstitial spaces and alveoli, culminating in ARDS. The damage induced by neutrophils to the lung’s endothelial and epithelial barriers is deemed a primary contributor to ARDS.

HBP, a cytokine released by activated immune cells, has been implicated in inducing lung inflammation and oxidative stress. As a key component of granules in polymorphonuclear neutrophils (PMNs), HBP plays a pivotal role in initiating the cascade of inflammation and enhancing vascular permeability (10). Secreted by emigrating PMNs, HBP attaches to the endothelial glycocalyx, presenting itself to circulating leukocytes. It activates monocytes rolling along the endothelium, leading to stable monocyte arrest, adhesion, transendothelial extravasation, and targeted migration to injury sites. HBP also triggers a swift increase in cytosolic free Ca^2+^ in adjacent endothelial cells, the formation of actin stress fibers, and heightened paracellular permeability (11, 12). Immunoneutralization of HBP in neutrophil-derived secretions effectively inhibits these activities, underscoring HBP’s crucial role in mediating neutrophil-induced changes in vascular permeability.

In this study, HBP levels surged and were closely linked with the lung wet/dry ratio and BALF protein levels, reflecting the compromised alveolar-capillary barrier that facilitates leukocyte migration and protein influx. The results are consistent with the changes in vascular permeability in the pathophysiology of ARDS. Numerous clinical studies have explored the relationship between HBP and ARDS. HBP is posited as a potential mediator of sepsis-induced ALI through its impact on endothelial permeability (13). In ARDS research, patients have displayed significantly elevated plasma HBP levels compared to those with cardiogenic pulmonary edema, with HBP serving as a potent prognostic marker for short-term mortality and hypoxemia severity (14). Moreover, in studies on transfusion-related ALI, PMNs were shown to release substantial HBP amounts in response to human antibodies, without concurrent interleukin-6 or tumor necrosis factor-alpha release, highlighting HBP’s role as a primary effector molecule in such cases (8). Our previous work has indicated that early-stage HBP elevation is a reliable biomarker for predicting sepsis-associated ARDS onset (9).

HBP’s association with infection has been well-documented, with plasma HBP levels serving as a robust indicator for distinguishing severe sepsis with circulatory failure from less severe infections (15). However, investigations into HBP levels in body fluids other than plasma have been scarce. A multicenter study in Sweden on the diagnostic and predictive value of HBP in urinary tract infections found HBP to be an effective diagnostic marker, suggesting its potential utility in identifying adult patients with suspected UTIs (16). Considering BALF as a marker of pulmonary exudation due to vascular leakage, its HBP levels are likely closely linked to lung injury severity. Previous ARDS research has predominantly focused on plasma HBP as a marker of systemic inflammation, with little attention to BALF HBP levels directly from the lungs. This study aimed to fill that gap by evaluating BALF HBP levels in both animal models and patients with ALI/ARDS.

In our animal studies, we utilized the lung wet/dry ratio and BALF protein levels as indicators of lung injury severity. Both plasma and BALF HBP levels were elevated in lung injury models compared to controls, with injury severity positively correlating with BALF HBP levels. Clinical findings revealed higher BALF and plasma HBP levels in ARDS patients versus those with cardiogenic pulmonary edema, establishing a significant correlation between BALF HBP, plasma HBP, and ARDS severity.

These findings underscore that BALF HBP levels in ARDS patients are markedly higher than in cases of cardiogenic pulmonary edema due to elevated hydrostatic pressure. The concurrent elevation of HBP in BALF and plasma aligns with the pathological mechanisms of pulmonary neutrophil aggregation and HBP release, leading to vascular leakage and impaired pulmonary oxygen exchange in ARDS. Thus, lung-derived HBP levels appear more intimately linked to lung injury severity, offering insights into the pathophysiological processes underlying ARDS.

### 4.1 Limitations

1) This study was conducted at a single institution and involved a relatively small cohort. To further substantiate these findings, larger-scale studies encompassing a greater number of participants are necessary.
2) While our study identified elevated HBP levels in BALF, the precise mechanisms driving this increase require clarification through further basic research.

## 5 Conclusion

In summary, our investigation reveals that HBP levels in bronchoalveolar lavage fluid (BALF) are markedly elevated in both animal models and humans experiencing lung injury, displaying a significant correlation with the injury’s severity. Notably, BALF HBP demonstrated a stronger association with lung injury compared to plasma HBP. These findings enhance our understanding of HBP’s pathological significance in acute respiratory distress syndrome (ARDS) and lay a theoretical foundation for targeted interventions aimed at modulating HBP levels in the lungs to mitigate or prevent ARDS.

## Data Availability

The original contributions presented in the study are included in the article/supplementary material, further inquiries can be directed to the corresponding author.
